# Fibroblasts, myofibroblasts and cardiac arrhythmias

**DOI:** 10.20517/jca.2023.37

**Published:** 2023-10-25

**Authors:** Nikolaos G. Frangogiannis

**Affiliations:** The Wilf Family Cardiovascular Research Institute, Department of Medicine and Department of Microbiology and Immunology, Albert Einstein College of Medicine, Bronx, NY 10461, USA.

Fibrosis accompanies most cardiac pathologic conditions and may contribute to the development of ventricular dysfunction and the pathogenesis of dysrhythmias^[1]^. Traditional concepts suggest that the role of fibrotic remodeling in arrhythmogenesis involves the increased deposition of collagens in the cardiac interstitium that may disrupt cardiomyocyte connectivity^[2]^. Over the last 20 years, *in vitro* evidence and some associative *in vivo* studies have suggested an additional mechanism of fibrosis-associated arrhythmia generation, resulting from the electrical coupling of fibroblasts and myofibroblasts with cardiomyocytes through the formation of gap junctions^[3–6]^. However, the *in vivo* role of gap junctional fibroblast-cardiomyocyte coupling has remained controversial^[7]^. It has been suggested that heterocellular fibroblast-cardiomyocyte coupling may represent a cell culture artifact and its potential *in vivo* role in arrhythmogenesis has not been demonstrated.

In a recent study by the Deb laboratory published in *Science*, Wang *et al.* provide the first *in vivo* evidence demonstrating a role for scar fibroblasts in cardiac excitability and induction of arrhythmias in myocardial infarction^[8]^. The authors used an impressive and elegant combination of *in vivo* mouse model studies, *in vitro* experiments, and mathematical modeling to study the pathophysiologic relevance and molecular basis of fibroblast-cardiomyocyte coupling in the infarcted heart. First, they engineered a transgenic mouse with fibroblast-specific expression of the light-sensitive cationic channel channelrhodopsin-2 (ChR2), thus enabling induction of membrane depolarization exclusively in fibroblasts upon optical stimulation with blue light. Using this transgenic optogenetic mouse, they demonstrated that optical stimulation of the scar tissue 10 days after myocardial infarction elicited organ-wide electrical activity and triggered ventricular arrhythmias. In contrast, optical stimulation of the uninjured area had no pro-arrhythmic effects. Coupling between fibroblasts and cardiomyocytes and subsequent arrhythmia generation were not dependent on the main cardiac gap junctional protein connexin 43. A combination of *in vitro* studies and mathematical modeling suggested that heterocellular fibroblast-cardiomyocyte coupling may result from synergistic actions between gap junctional proteins and a (rather enigmatic) ephaptic non-gap junctional mechanism^[9]^ that involves the generation of electrical fields in the interstitial space between the coupled cells without the direct transfer of ions.

The study shows for the first time that fibroblast-cardiomyocyte coupling can generate arrhythmias in the infarcted heart. Although the documentation of fibroblast-driven arrhythmia generation is a major advance in cellular electrophysiology, whether heterocellular coupling between interstitial cells and cardiomyocytes is implicated in the generation of arrhythmias in injured mammalian hearts remains unknown. As with all high-quality studies, the current investigation not only addresses an important question, but also raises several intriguing questions [Figure 1].

## DOES FIBROBLAST-CARDIOMYOCYTE COUPLING MEDIATE ARRHYTHMIAS AFTER CARDIAC INJURY?

Wang *et al.* used a model of exogenous optically-stimulated depolarization of all scar fibroblasts to support the notion that fibroblast-cardiomyocyte coupling can generate arrhythmias^[8]^. However, this experimental model does not examine whether coupling involving the fibroblasts plays a role in the generation of arrhythmias, which are commonly noted in infarcted, injured, and remodeling hearts. The well-documented close spatial relation between activated fibroblasts and cardiomyocytes in the infarct border zone and in the remodeling myocardium supports the plausibility of this concept. However, whether fibroblast populations exhibit spontaneous depolarization and cardiomyocyte coupling in infarcted hearts is unknown. Even if small populations of fibroblasts depolarize, whether this is sufficient to trigger an arrhythmic response is unclear. The optogenetic model used in the current study induced simultaneous depolarization of all scar fibroblasts, which is unlikely to occur in the infarcted or remodeling heart^[10]^. Experiments studying the effects of fibroblast-specific interventions that block fibroblast-cardiomyocyte coupling on the incidence of arrhythmias in infarcted or failing hearts are required to address these questions. Such experiments pose major challenges. Identification of the molecular basis of heterocellular coupling would be required, and analysis would be greatly limited by the challenges in studying post-infarction arrhythmias in the mouse.

## DOES ARRHYTHMOGENICITY INVOLVE SPECIFIC FIBROBLAST SUBPOPULATIONS?

Fibroblast populations exhibit remarkable heterogeneity and are composed of several cell types with distinct profiles and functional properties^[11]^. The relative capacity of various fibroblast subtypes to couple with cardiomyocytes and initiate arrhythmias is not known. *In vitro* co-culture studies have suggested that myofibroblasts^[12]^, the activated fibroblasts that express contractile proteins such as a-smooth muscle actin (a-SMA) and infiltrate healing infarcts, may have increased arrhythmogenic potential that is attenuated upon pharmacologic ablation of the a-SMA-containing stress fibers^[13]^. In the current study, optical stimulation of normal hearts or of the remote non-infarcted myocardium did not result in changes in heart rate and did not trigger arrhythmias. Although the finding may simply reflect the much higher numbers of fibroblasts in the scar, it is possible that infarct myofibroblasts (or another subpopulation of activated infarct fibroblasts) may have unique electrophysiologic properties that enhance depolarization, or foster coupling with cardiomyocytes.

## DOES FIBROBLAST-CARDIOMYOCYTE COUPLING CONTRIBUTE TO ARRHYTHMOGENESIS IN CHRONIC HEART FAILURE?

Fibroblasts are highly dynamic cells and undergo dramatic phenotypic transitions during the phases of infarct healing^[14]^. As the scar matures, infarct fibroblasts disassemble the a-SMA expressing stress fibers and convert to matrifibrocytes, which are specialized connective tissue cells with matrix-preserving properties^[15]^. In order to understand the potential involvement of fibroblast-driven arrhythmogenesis in chronic post-infarction heart failure, it is important to examine whether fibroblast-cardiomyocyte coupling persists in mature scars. Intuitively, the extensive deposition of cross-linked collagen in mature scars may perturb coupling while enhancing arrhythmogenesis by blocking conduction. In the current study, Wang *et al.* focused predominantly on the proliferative phase of infarct healing (10 days after infarction), which is associated with abundant myofibroblast infiltration^[8]^. However, they also examined arrhythmogenicity 12 weeks after infarction, at a time point characterized by relative depletion of myofibroblasts and extensive deposition of cross-linked extracellular matrix. At this time point, optical stimulation of fibroblast depolarization triggered asystole in a subset of the animals. This was attributed to rapid repetitive ventricular activation from fibroblast depolarization that caused high-grade atrioventricular conduction block in the absence of a ventricular escape rhythm. The cross-linked matrix environment and the phenotype of the fibroblasts in the mature scar may perturb conduction, favoring atrioventricular block.

In addition, the study does not address the potential involvement of fibroblast-cardiomyocyte coupling in the increased arrhythmic burden associated with chronic non-ischemic heart failure. Fibroblasts undergo activation in a broad range of conditions, including cardiomyopathies associated with pressure overload, genetic diseases, or metabolic dysregulation. The distinct fibroblast profiles associated with each one of these conditions may also exhibit differences in their capacity to couple with cardiomyocytes.

## WHAT IS THE MOLECULAR CIRCUITRY OF HETEROCELLULAR FIBROBLAST-CARDIOMYOCYTE COUPLING?

Despite the use of an impressive range of elegant experimental approaches, Wang *et al.* were not able to dissect the specific molecular mechanisms involved in fibroblast-cardiomyocyte coupling and subsequent arrhythmia generation^[8]^. *In vivo*, connexin 43 loss did not affect coupling, organ excitation, and arrhythmia generation. *In vitro*, fibroblast deletion of other gap junctional proteins, including *Cx45, Cx40*, and *Panx1*, had no effects on coupling in a co-culture model with neonatal cardiomyocytes. Thus, the mechanism of coupling remains enigmatic and may involve both gap-junctional and ephaptic interactions.

## CONCLUSIONS

The work by Wang *et al.* demonstrates the power of transgenic mouse models and innovative experimental techniques in addressing important mechanistic questions^[8]^. The authors demonstrated for the first time that fibroblast-cardiomyocyte coupling can induce arrhythmia generation *in vivo*. However, all experimental studies have limitations. Although the findings document that fibroblasts can couple with cardiomyocytes and generate arrhythmias, whether this mechanism plays a significant role in arrhythmogenesis associated with common pathologic conditions (such as myocardial infarction and heart failure) is unknown. Considering the limitations of the mouse model in recapitulating human arrhythmogenesis, convincing documentation of the significance of this mechanism may pose major challenges. However, the study has a major impact on our evolving paradigm of arrhythmia generation, emphasizing that fibroblast-driven arrhythmogenesis does not only involve deposition of extracellular matrix proteins and subsequent conduction block, but may also be mediated through coupling and direct stimulation of cardiomyocyte excitability.

## Figures and Tables

**Figure 1.**
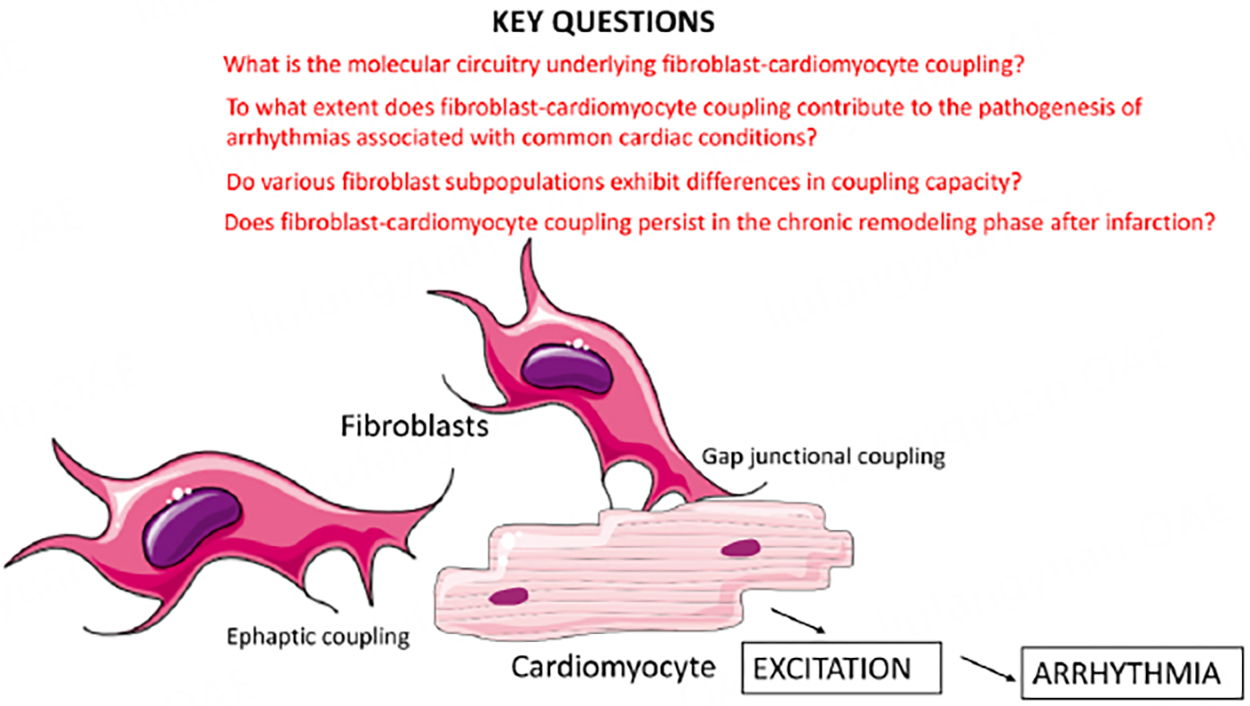
Fibroblast-driven arrhythmogenesis in myocardial infarction. A recently published study by Wang *et al.* documented for the first time that fibroblasts in infarcted hearts can couple with cardiomyocytes and trigger arrhythmias^[8]^. Thus, fibrosis can promote arrhythmogenesis not only through the conduction-blocking effects of the extracellular matrix deposited between cardiomyocytes, but also through direct fibroblast-mediated cardiomyocyte excitation. Coupling may involve both gap junctional and ephaptic coupling. The study also raises several intriguing questions for further investigation.
